# (Diamino­methyl­idene)sulfonium chloride–thio­urea (3/2)

**DOI:** 10.1107/S1600536811052809

**Published:** 2012-01-07

**Authors:** Hafid Zouihri

**Affiliations:** a Laboratoire Privé de Cristallographie (L.P.C), Kénitra, Morocco.

## Abstract

The asymetric unit of the title salt, 3CH_5_N_2_S^+^·3Cl^−^·2CH_4_N_2_S, contains two mol­ecules of thio­urea, three (diamino­methyl­idene)sulfonium cations and three chloride anions. The crystal packing is stabilized by N—H⋯Cl, N—H⋯S, S—H⋯Cl and S—H⋯S hydrogen bonds, forming a three-dimensional network.

## Related literature

For applications of thio­urea salts, see: Xing *et al.* (1987 ▶); Velsko *et al.* (1990 ▶).
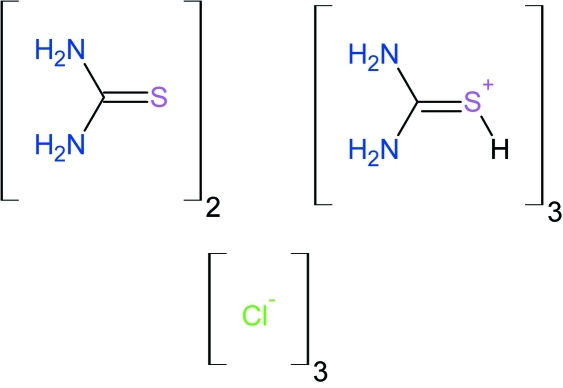


## Experimental

### 

#### Crystal data

3CH_5_N_2_S^+^·3Cl^−^·2CH_4_N_2_S
*M* *_r_* = 489.98
Monoclinic, 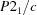

*a* = 16.3469 (6) Å
*b* = 8.9579 (3) Å
*c* = 16.1505 (5) Å
β = 109.105 (2)°
*V* = 2234.72 (13) Å^3^
*Z* = 4
Mo *K*α radiation
μ = 0.89 mm^−1^
*T* = 100 K
0.45 × 0.32 × 0.29 mm

#### Data collection

Bruker APEXII CCD detector diffractometer
25994 measured reflections
4885 independent reflections
4190 reflections with *I* > 2σ(*I*)
*R*_int_ = 0.036

#### Refinement

*R*[*F* ^2^ > 2σ(*F* ^2^)] = 0.026
*wR*(*F* ^2^) = 0.068
*S* = 1.06
4885 reflections
300 parameters
15 restraints
All H-atom parameters refined
Δρ_max_ = 0.45 e Å^−3^
Δρ_min_ = −0.39 e Å^−3^

### 

Data collection: *APEX2* (Bruker, 2005 ▶); cell refinement: *SAINT* (Bruker, 2005 ▶); data reduction: *SAINT*; program(s) used to solve structure: *SHELXS97* (Sheldrick, 2008 ▶); program(s) used to refine structure: *SHELXL97* (Sheldrick, 2008 ▶); molecular graphics: *PLATON* (Spek, 2009 ▶); software used to prepare material for publication: *publCIF* (Westrip, 2010 ▶).

## Supplementary Material

Crystal structure: contains datablock(s) I, global. DOI: 10.1107/S1600536811052809/bt5746sup1.cif

Structure factors: contains datablock(s) I. DOI: 10.1107/S1600536811052809/bt5746Isup2.hkl

Supplementary material file. DOI: 10.1107/S1600536811052809/bt5746Isup3.cml

Additional supplementary materials:  crystallographic information; 3D view; checkCIF report

## Figures and Tables

**Table 1:** Hydrogen-bond geometry (Å, °)

*D*—H⋯*A*	*D*—H	H⋯*A*	*D*⋯*A*	*D*—H⋯*A*
N1—H1*N*⋯S3	0.82 (2)	2.76 (2)	3.5403 (17)	161.9 (18)
N1—H2*N*⋯Cl1	0.848 (18)	2.474 (18)	3.2683 (16)	156.2 (18)
N10—H17*N*⋯Cl3^i^	0.86 (2)	2.45 (2)	3.2261 (17)	152 (2)
N10—H18*N*⋯Cl1^ii^	0.85 (2)	2.35 (2)	3.2019 (14)	175 (2)
N2—H3*N*⋯Cl1	0.815 (18)	2.624 (18)	3.3656 (16)	152.0 (17)
N2—H4*N*⋯Cl1^ii^	0.86 (2)	2.45 (2)	3.3060 (17)	174.5 (17)
N3—H5*N*⋯Cl2	0.86 (2)	2.51 (2)	3.3333 (16)	161 (2)
N3—H6*N*⋯Cl1^iii^	0.86 (2)	2.47 (2)	3.2799 (16)	157 (2)
N4—H7*N*⋯Cl3	0.84 (2)	2.43 (2)	3.2422 (17)	163 (2)
N4—H8*N*⋯Cl1^iii^	0.84 (2)	2.57 (2)	3.3327 (16)	151 (2)
N4—H8*N*⋯S1^iv^	0.84 (2)	2.83 (2)	3.3571 (16)	123 (2)
N5—H9*N*⋯S1	0.85 (2)	2.62 (2)	3.4493 (18)	166 (2)
N5—H10*N*⋯Cl2^v^	0.91 (2)	2.35 (2)	3.2262 (16)	161 (2)
N6—H11*N*⋯Cl2^vi^	0.88 (2)	2.47 (2)	3.3555 (17)	176 (1)
N6—H12*N*⋯Cl2^v^	0.88 (2)	2.65 (2)	3.4328 (15)	149 (2)
N7—H13*N*⋯Cl2	0.86 (2)	2.37 (2)	3.2219 (15)	177 (1)
N7—H14*N*⋯Cl3^vii^	0.85 (2)	2.42 (2)	3.2066 (17)	154 (2)
N8—H15*N*⋯S3^viii^	0.85 (2)	2.44 (2)	3.2649 (16)	164 (2)
N8—H16*N*⋯Cl3^vii^	0.87 (2)	2.39 (2)	3.2036 (17)	156 (2)
N9—H19*N*⋯Cl3^i^	0.84 (2)	2.38 (2)	3.1674 (17)	157 (2)
N9—H20*N*⋯S1^vii^	0.872 (19)	2.38 (2)	3.2412 (16)	167.7 (19)
S2—H2*S*⋯Cl2	1.253 (19)	2.315 (19)	3.5612 (6)	172.9 (14)
S4—H4*S*⋯S3^viii^	1.24 (2)	2.69 (2)	3.8755 (8)	159.2 (15)
S5—H5*S*⋯S1^vii^	1.29 (2)	2.64 (2)	3.8691 (7)	159.3 (16)

Symmetry codes: (i) 
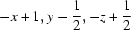
; (ii) 
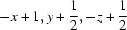
; (iii) 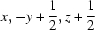; (iv) 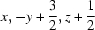; (v) 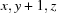; (vi) 
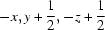
; (vii) 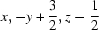; (viii) 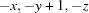.

**Fig. 1.**
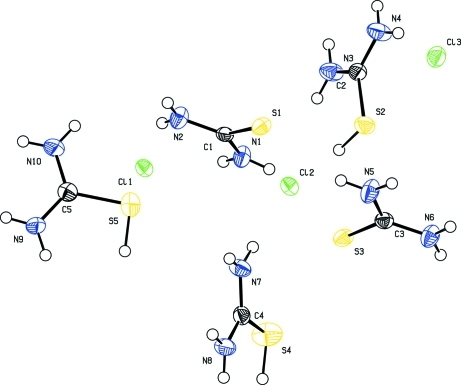
Molecular view of the title compound showing the atom-labeling scheme. Displace- ment ellipsoids are drawn at the 50% probability level. H atoms are represented as small spheres of arbitrary radii.

**Fig. 2.**
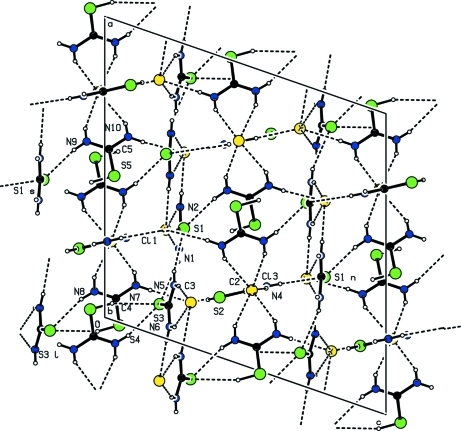
Projection of the title compound along the c axis, H-bonds are represented by dashed lines.

## supplementary crystallographic information

### Comment

Various semi-organic NLO materials of thiourea were found to have higher mechanical strentgh, chemical stability, large nonlinearity, high resistance to laser induced damage, low angular sensitivity and good mechanical hardness [Xing, *et al.* 1987 and Velsko, *et al.* 1990]. Herein we present the crystal structure of the title compound (I).

The assymetric unit of the title salt compound contains two molecules of thiourea (A and B), three cations of (diaminomethylidene)sulfonium (C, D and E) and three chloride anions.

The dihedral angles between the five molecules of the asymmetric unit are: A/B = 11,96 (9)°, A/C = 75,67 (9)°, A/D = 84,56 (9)°, A/E = 85,02 (9)°, B/C = 71.4 (3)°, B/D = 88,34 (9)° and B/E = 73,21 (9)° (Fig. 1).

In the crystal, the components are linked by a combination of thirteen N—H···Cl, five N—H···S, one S—H···Cl and two S—H···S hydrogen bonds into a three-dimensional structure. (Fig. 2 and Table. 1).

### Experimental

The title compound was synthetized at ambient temperature by a mixture of 5 mmol s of thiourea and 5 mmol of HCl in ethanolic solution. The solution was slowly evaporated until solvent completely dried and white crystalline salt was obtained.

Suitable transparent crystals for X-ray mesearment were grown from the final product in aqueous solution by slow evaporation method.

### Refinement

All H atoms were located from difference Fourier maps and refined isotrpically, with restained distance N—H = 0.88 (0.02) A.

The highest residual density was found 0.82 A from S4 and the deepest hole 0.74 A from S4.

### Crystal data

**Table:**

3CH_5_N_2_S^+^·3Cl^−^·2CH_4_N_2_S	*F*(000) = 1016
*M**_r_* = 489.98	*D*_x_ = 1.456 Mg m^−^^3^
Monoclinic, *P*2_1_/*c*	Mo *K*α radiation, λ = 0.71073 Å
Hall symbol: -P 2ybc	Cell parameters from 257 reflections
*a* = 16.3469 (6) Å	θ = 1.9–26.7°
*b* = 8.9579 (3) Å	µ = 0.89 mm^−^^1^
*c* = 16.1505 (5) Å	*T* = 100 K
β = 109.105 (2)°	Prism, colourless
*V* = 2234.72 (13) Å^3^	0.45 × 0.32 × 0.29 mm
*Z* = 4	

### Data collection

**Table:**

Bruker APEXII CCD detector diffractometer	4190 reflections with *I* > 2σ(*I*)
Radiation source: fine-focus sealed tube	*R*_int_ = 0.036
graphite	θ_max_ = 27.0°, θ_min_ = 1.3°
ω and φ scans	*h* = −19→20
25994 measured reflections	*k* = −11→11
4885 independent reflections	*l* = −20→20

### Refinement

**Table:**

Refinement on *F*^2^	Primary atom site location: structure-invariant direct methods
Least-squares matrix: full	Secondary atom site location: difference Fourier map
*R*[*F*^2^ > 2σ(*F*^2^)] = 0.026	Hydrogen site location: inferred from neighbouring sites
*wR*(*F*^2^) = 0.068	All H-atom parameters refined
*S* = 1.06	*w* = 1/[σ^2^(*F*_o_^2^) + (0.030*P*)^2^ + 0.6051*P*] where *P* = (*F*_o_^2^ + 2*F*_c_^2^)/3
4885 reflections	(Δ/σ)_max_ = 0.001
300 parameters	Δρ_max_ = 0.45 e Å^−^^3^
15 restraints	Δρ_min_ = −0.39 e Å^−^^3^

### Special details

**Table:**

Geometry. All e.s.d.'s (except the e.s.d. in the dihedral angle between two l.s. planes) are estimated using the full covariance matrix. The cell e.s.d.'s are taken into account individually in the estimation of e.s.d.'s in distances, angles and torsion angles; correlations between e.s.d.'s in cell parameters are only used when they are defined by crystal symmetry. An approximate (isotropic) treatment of cell e.s.d.'s is used for estimating e.s.d.'s involving l.s. planes.
Refinement. Refinement of *F*^2^ against ALL reflections. The weighted *R*-factor *wR* and goodness of fit *S* are based on *F*^2^, conventional *R*-factors *R* are based on *F*, with *F* set to zero for negative *F*^2^. The threshold expression of *F*^2^ > σ(*F*^2^) is used only for calculating *R*-factors(gt) *etc*. and is not relevant to the choice of reflections for refinement. *R*-factors based on *F*^2^ are statistically about twice as large as those based on *F*, and *R*- factors based on ALL data will be even larger.

### Fractional atomic coordinates and isotropic or equivalent isotropic displacement parameters (Å^2^)

**Table:**

	*x*	*y*	*z*	*U*_iso_*/*U*_eq_	
C1	0.38413 (9)	0.58264 (16)	0.27012 (9)	0.0213 (3)	
C2	0.25156 (10)	0.52767 (18)	0.50586 (9)	0.0248 (3)	
C3	0.11263 (10)	0.86257 (17)	0.22690 (10)	0.0243 (3)	
C4	0.07611 (10)	0.32825 (17)	0.03972 (10)	0.0256 (3)	
C5	0.56391 (11)	0.66084 (17)	0.01498 (10)	0.0274 (3)	
Cl1	0.36083 (2)	0.16969 (4)	0.21733 (2)	0.02368 (9)	
Cl2	0.14816 (2)	0.26513 (4)	0.30590 (2)	0.02530 (9)	
Cl3	0.25954 (3)	0.95922 (5)	0.52321 (3)	0.03198 (10)	
H10N	0.1886 (13)	1.0337 (17)	0.2623 (13)	0.049 (6)*	
H11N	−0.0063 (10)	0.887 (2)	0.2194 (12)	0.039 (5)*	
H12N	0.0483 (13)	1.0283 (18)	0.2478 (13)	0.047 (6)*	
H13N	0.1429 (12)	0.326 (2)	0.1616 (10)	0.038 (5)*	
H14N	0.1828 (11)	0.408 (2)	0.1057 (12)	0.039 (5)*	
H15N	0.0337 (12)	0.357 (2)	−0.0832 (11)	0.045 (6)*	
H16N	0.1202 (11)	0.429 (2)	−0.0372 (12)	0.039 (5)*	
H17N	0.6669 (11)	0.561 (2)	0.0740 (13)	0.044 (6)*	
H18N	0.6263 (13)	0.628 (2)	0.1350 (11)	0.045 (6)*	
H19N	0.6042 (11)	0.582 (2)	−0.0693 (12)	0.042 (6)*	
H1N	0.2689 (14)	0.552 (2)	0.2609 (13)	0.048 (6)*	
H20N	0.5209 (13)	0.667 (2)	−0.1093 (12)	0.035 (5)*	
H2N	0.3116 (12)	0.413 (2)	0.2562 (12)	0.034 (5)*	
H2S	0.1772 (14)	0.501 (2)	0.3670 (14)	0.063 (7)*	
H3N	0.4498 (11)	0.418 (2)	0.2606 (11)	0.028 (5)*	
H4N	0.5001 (12)	0.556 (2)	0.2727 (12)	0.033 (5)*	
H4S	−0.0549 (15)	0.234 (2)	−0.0260 (15)	0.065 (7)*	
H5N	0.2310 (11)	0.331 (2)	0.4620 (10)	0.033 (5)*	
H5S	0.4337 (15)	0.768 (2)	−0.0474 (15)	0.068 (7)*	
H6N	0.2823 (13)	0.341 (2)	0.5582 (11)	0.052 (6)*	
H7N	0.2821 (12)	0.7027 (17)	0.5739 (12)	0.036 (5)*	
H8N	0.3128 (12)	0.567 (2)	0.6237 (10)	0.042 (6)*	
H9N	0.2319 (10)	0.890 (2)	0.2464 (12)	0.038 (5)*	
N1	0.31308 (10)	0.50667 (17)	0.26422 (10)	0.0286 (3)	
N10	0.62646 (10)	0.61272 (18)	0.08312 (9)	0.0349 (3)	
N2	0.45314 (10)	0.50807 (17)	0.26805 (10)	0.0295 (3)	
N3	0.25464 (10)	0.38179 (17)	0.50862 (10)	0.0341 (3)	
N4	0.28862 (11)	0.60913 (18)	0.57553 (10)	0.0362 (4)	
N5	0.18693 (10)	0.93523 (17)	0.24771 (11)	0.0355 (3)	
N6	0.04351 (10)	0.93486 (16)	0.23118 (10)	0.0320 (3)	
N7	0.14216 (10)	0.35255 (17)	0.11049 (9)	0.0307 (3)	
N8	0.07441 (10)	0.37960 (17)	−0.03649 (9)	0.0307 (3)	
N9	0.56308 (10)	0.63381 (18)	−0.06456 (9)	0.0317 (3)	
S1	0.38657 (3)	0.77282 (4)	0.28160 (3)	0.02625 (10)	
S2	0.19881 (3)	0.62081 (5)	0.40844 (3)	0.03084 (11)	
S3	0.10697 (3)	0.67952 (4)	0.19506 (3)	0.02933 (10)	
S4	−0.00958 (3)	0.22533 (6)	0.05196 (4)	0.04788 (14)	
S5	0.48080 (3)	0.76310 (6)	0.03389 (3)	0.04573 (13)	

### Atomic displacement parameters (Å^2^)

**Table:**

	*U*^11^	*U*^22^	*U*^33^	*U*^12^	*U*^13^	*U*^23^
C1	0.0220 (8)	0.0232 (7)	0.0164 (7)	0.0000 (6)	0.0031 (6)	−0.0008 (5)
C2	0.0248 (8)	0.0286 (8)	0.0211 (8)	0.0015 (6)	0.0077 (6)	0.0016 (6)
C3	0.0261 (8)	0.0229 (7)	0.0215 (7)	0.0010 (6)	0.0045 (6)	0.0007 (6)
C4	0.0254 (8)	0.0247 (8)	0.0275 (8)	−0.0002 (6)	0.0099 (7)	−0.0041 (6)
C5	0.0271 (9)	0.0276 (8)	0.0276 (8)	−0.0040 (7)	0.0093 (7)	−0.0006 (6)
Cl1	0.0267 (2)	0.02205 (18)	0.02225 (18)	−0.00058 (14)	0.00803 (15)	−0.00263 (13)
Cl2	0.0296 (2)	0.02340 (18)	0.02352 (19)	−0.00071 (15)	0.00952 (16)	−0.00253 (14)
Cl3	0.0266 (2)	0.0341 (2)	0.0364 (2)	0.00110 (16)	0.01196 (17)	−0.00136 (17)
N1	0.0235 (8)	0.0219 (7)	0.0418 (8)	−0.0027 (6)	0.0126 (6)	−0.0068 (6)
N10	0.0344 (9)	0.0496 (9)	0.0197 (7)	0.0044 (7)	0.0076 (6)	0.0011 (6)
N2	0.0250 (8)	0.0211 (7)	0.0433 (9)	−0.0008 (6)	0.0122 (6)	−0.0021 (6)
N3	0.0438 (10)	0.0277 (8)	0.0246 (8)	0.0012 (7)	0.0027 (7)	0.0029 (6)
N4	0.0482 (10)	0.0320 (8)	0.0220 (7)	−0.0004 (7)	0.0027 (7)	−0.0004 (6)
N5	0.0243 (8)	0.0257 (7)	0.0560 (10)	−0.0006 (6)	0.0124 (7)	−0.0089 (7)
N6	0.0255 (8)	0.0237 (7)	0.0463 (9)	0.0012 (6)	0.0113 (7)	−0.0020 (6)
N7	0.0317 (8)	0.0394 (8)	0.0207 (7)	−0.0071 (7)	0.0082 (6)	−0.0012 (6)
N8	0.0270 (8)	0.0418 (8)	0.0218 (7)	−0.0056 (7)	0.0060 (6)	−0.0018 (6)
N9	0.0287 (8)	0.0426 (9)	0.0212 (7)	0.0079 (7)	0.0047 (6)	0.0034 (6)
S1	0.0223 (2)	0.01955 (19)	0.0319 (2)	−0.00003 (15)	0.00212 (16)	−0.00217 (15)
S2	0.0373 (2)	0.0297 (2)	0.0220 (2)	0.00586 (17)	0.00489 (17)	0.00139 (16)
S3	0.0255 (2)	0.02236 (19)	0.0326 (2)	0.00159 (15)	−0.00071 (17)	−0.00544 (16)
S4	0.0410 (3)	0.0524 (3)	0.0473 (3)	−0.0191 (2)	0.0104 (2)	0.0089 (2)
S5	0.0439 (3)	0.0502 (3)	0.0446 (3)	0.0125 (2)	0.0165 (2)	−0.0067 (2)

### Geometric parameters (Å, °)

**Table:**

C1—N1	1.322 (2)	N4—H8N	0.841 (15)
C1—N2	1.321 (2)	N5—H10N	0.911 (15)
C2—N4	1.311 (2)	N5—H9N	0.847 (14)
C2—N3	1.308 (2)	N6—H11N	0.884 (14)
C3—N6	1.324 (2)	N6—H12N	0.875 (15)
C3—N5	1.321 (2)	N7—H14N	0.851 (14)
C4—N7	1.308 (2)	N7—H13N	0.855 (14)
C4—N8	1.306 (2)	N8—H16N	0.873 (14)
C5—N10	1.306 (2)	N8—H15N	0.851 (15)
C5—N9	1.303 (2)	N9—H19N	0.840 (15)
N1—H1N	0.81 (2)	N9—H20N	0.87 (2)
N1—H2N	0.84 (2)	S1—C1	1.7127 (15)
N10—H18N	0.850 (15)	S2—H2S	1.25 (2)
N10—H17N	0.857 (15)	S2—C2	1.7398 (15)
N2—H4N	0.861 (19)	S3—C3	1.7120 (16)
N2—H3N	0.811 (19)	S4—H4S	1.24 (2)
N3—H6N	0.862 (15)	S4—C4	1.7414 (16)
N3—H5N	0.855 (14)	S5—H5S	1.29 (2)
N4—H7N	0.844 (15)	S5—C5	1.7464 (17)
			
C2—S2—H2S	92.4 (10)	C1—N2—H3N	119.1 (13)
C5—S5—H5S	94.6 (10)	C1—N2—H4N	119.4 (12)
C4—S4—H4S	94.9 (10)	H3N—N2—H4N	121.4 (18)
N2—C1—N1	118.37 (15)	C3—N5—H9N	119.5 (13)
N2—C1—S1	121.06 (12)	C3—N5—H10N	119.3 (13)
N1—C1—S1	120.56 (12)	H9N—N5—H10N	121.2 (18)
N8—C4—N7	121.45 (15)	C4—N7—H13N	123.0 (13)
N8—C4—S4	121.58 (13)	C4—N7—H14N	117.6 (13)
N7—C4—S4	116.97 (12)	H13N—N7—H14N	118.9 (18)
N9—C5—N10	121.48 (16)	C1—N1—H2N	120.1 (12)
N9—C5—S5	120.81 (13)	C1—N1—H1N	119.4 (15)
N10—C5—S5	117.71 (13)	H2N—N1—H1N	119.9 (19)
N5—C3—N6	118.38 (15)	C5—N9—H20N	120.2 (12)
N5—C3—S3	120.21 (12)	C5—N9—H19N	116.2 (13)
N6—C3—S3	121.41 (13)	H20N—N9—H19N	123.6 (18)
N3—C2—N4	121.66 (15)	C3—N6—H12N	119.5 (14)
N3—C2—S2	120.80 (12)	C3—N6—H11N	119.5 (12)
N4—C2—S2	117.53 (13)	H12N—N6—H11N	120.9 (18)
C2—N3—H5N	119.8 (13)	C5—N10—H17N	117.8 (13)
C2—N3—H6N	117.5 (14)	C5—N10—H18N	121.4 (14)
H5N—N3—H6N	123 (2)	H17N—N10—H18N	120.7 (19)
C2—N4—H8N	119.2 (14)	C4—N8—H15N	121.5 (14)
C2—N4—H7N	120.5 (13)	C4—N8—H16N	115.9 (13)
H8N—N4—H7N	119.7 (19)	H15N—N8—H16N	122.2 (18)

### Hydrogen-bond geometry (Å, °)

**Table:**

*D*—H···*A*	*D*—H	H···*A*	*D*···*A*	*D*—H···*A*
N1—H1N···S3	0.82 (2)	2.76 (2)	3.5403 (17)	161.9 (18)
N1—H2N···Cl1	0.848 (18)	2.474 (18)	3.2683 (16)	156.2 (18)
N10—H17N···Cl3^i^	0.86 (2)	2.44 (2)	3.2261 (17)	152 (2)
N10—H18N···Cl1^ii^	0.85 (2)	2.35 (2)	3.2019 (14)	175 (2)
N2—H3N···Cl1	0.815 (18)	2.624 (18)	3.3656 (16)	152.0 (17)
N2—H4N···Cl1^ii^	0.86 (2)	2.45 (2)	3.3060 (17)	174.5 (17)
N3—H5N···Cl2	0.86 (2)	2.51 (2)	3.3333 (16)	161 (2)
N3—H6N···Cl1^iii^	0.86 (2)	2.47 (2)	3.2799 (16)	157 (2)
N4—H7N···Cl3	0.84 (2)	2.43 (2)	3.2422 (17)	163 (2)
N4—H8N···Cl1^iii^	0.84 (2)	2.57 (2)	3.3327 (16)	151 (2)
N4—H8N···S1^iv^	0.84 (2)	2.83 (2)	3.3571 (16)	123 (2)
N5—H9N···S1	0.85 (2)	2.62 (2)	3.4493 (18)	166 (2)
N5—H10N···Cl2^v^	0.91 (2)	2.35 (2)	3.2262 (16)	161 (2)
N6—H11N···Cl2^vi^	0.88 (2)	2.47 (2)	3.3555 (17)	176 (1)
N6—H12N···Cl2^v^	0.88 (2)	2.65 (2)	3.4328 (15)	149 (2)
N7—H13N···Cl2	0.86 (2)	2.37 (2)	3.2219 (15)	177 (1)
N7—H14N···Cl3^vii^	0.85 (2)	2.42 (2)	3.2066 (17)	154 (2)
N8—H15N···S3^viii^	0.85 (2)	2.44 (2)	3.2649 (16)	164 (2)
N8—H16N···Cl3^vii^	0.87 (2)	2.38 (2)	3.2036 (17)	156 (2)
N9—H19N···Cl3^i^	0.84 (2)	2.38 (2)	3.1674 (17)	157 (2)
N9—H20N···S1^vii^	0.872 (19)	2.38 (2)	3.2412 (16)	167.7 (19)
S2—H2S···Cl2	1.253 (19)	2.315 (19)	3.5612 (6)	172.9 (14)
S4—H4S···S3^viii^	1.24 (2)	2.69 (2)	3.8755 (8)	159.2 (15)
S5—H5S···S1^vii^	1.29 (2)	2.64 (2)	3.8691 (7)	159.3 (16)

Symmetry codes: (i) −*x*+1, *y*−1/2, −*z*+1/2; (ii) −*x*+1, *y*+1/2, −*z*+1/2; (iii) *x*, −*y*+1/2, *z*+1/2; (iv) *x*, −*y*+3/2, *z*+1/2; (v) *x*, *y*+1, *z*; (vi) −*x*, *y*+1/2, −*z*+1/2; (vii) *x*, −*y*+3/2, *z*−1/2; (viii) −*x*, −*y*+1, −*z*.
